# Function, Structure, and Stability of Enzymes Confined in Agarose Gels

**DOI:** 10.1371/journal.pone.0086785

**Published:** 2014-01-21

**Authors:** Jeffrey Kunkel, Prashanth Asuri

**Affiliations:** Department of Bioengineering, Santa Clara University, Santa Clara, California, United States of America; Oak Ridge National Laboratory, United States of America

## Abstract

Research over the past few decades has attempted to answer how proteins behave in molecularly confined or crowded environments when compared to dilute buffer solutions. This information is vital to understanding *in vivo* protein behavior, as the average spacing between macromolecules in the cell cytosol is much smaller than the size of the macromolecules themselves. In our study, we attempt to address this question using three structurally and functionally different model enzymes encapsulated in agarose gels of different porosities. Our studies reveal that under standard buffer conditions, the initial reaction rates of the agarose-encapsulated enzymes are lower than that of the solution phase enzymes. However, the encapsulated enzymes retain a higher percentage of their activity in the presence of denaturants. Moreover, the concentration of agarose used for encapsulation had a significant effect on the enzyme functional stability; enzymes encapsulated in higher percentages of agarose were more stable than the enzymes encapsulated in lower percentages of agarose. Similar results were observed through structural measurements of enzyme denaturation using an 8-anilinonaphthalene-1-sulfonic acid fluorescence assay. Our work demonstrates the utility of hydrogels to study protein behavior in highly confined environments similar to those present *in vivo*; furthermore, the enhanced stability of gel-encapsulated enzymes may find use in the delivery of therapeutic proteins, as well as the design of novel strategies for biohybrid medical devices.

## Introduction

It is currently well established that traditional experimental conditions using dilute buffer solutions cannot accurately predict behavior of proteins in their *in vivo* cellular environment [1], [2]. Various macromolecules make up more than 60% of the cell cytosol creating a crowded or confined environment, with considerably low volumes of free water [1]. Consequently, the thermodynamics and kinetics of several biological processes in a living cell, such as rates of protein-catalyzed reactions, protein-protein interactions, and protein folding and unfolding can be expected to be quite different from those calculated using experimental conditions that use proteins in simple dilute buffers [3].

Recently, several studies have investigated protein behavior using *in vitro* conditions that more closely capture the *in vivo* cellular environment. Many of these studies have focused on the use of buffers containing high concentrations of natural and synthetic macromolecules, such as serum albumin, dextran, Ficolls, etc. [4]–[12]. Such macromolecularly crowded systems have been used to study kinetics of biochemical reactions and networks, as well as protein unfolding and refolding, and protein association and aggregation [4], [5], [8], [10], [12], [13]. Results from these studies have supported the hypothesis that the excluded volume effects arising in such crowded environments significantly impact protein fate and function [4], [7], [9], [11].

Fewer studies have reported the experimental investigations of molecular confinement on protein structure and function. *In vivo* confinement arises from encapsulation of proteins in spaces only moderately larger than the proteins (1–50 nm), e.g. ribosome tunnel or a chaperonin cavity. *In vitro* investigations of protein confinement have been achieved by encapsulating proteins in pores of silica sol-gels, polyacrylamide and agarose gels [14]–[16], or by sandwiching them between parallel zirconium layers [17], [18]. Eggers and Valentine reported encapsulation of proteins in silica using the sol-gel method and demonstrated significant enhancements in the thermal stability, possibly arising from the decreased flexibility of the proteins inside pores of the silica matrix [14]. Bismuto *et al.* used spectroscopic techniques and molecular dynamics simulations to explore the correlation between the structural dynamics and the function and stability of enzymes encapsulated in agarose gels [16]. Their study indicated that confinement in agarose gels limits the flexibility of the polypeptide backbone, thereby preventing protein unfolding at higher temperatures.

In this study, we investigated the effects of molecular confinement on enzyme kinetics, as well as their functional and structural stability under denaturing environments using hydrogel scaffolds with different porosities and, therefore, different degrees of confinement. Agarose was used to encapsulate the enzymes of interest because the pore size of agarose gels can be controlled by simply changing the concentration of agarose [19], [20]. We used two different concentrations of agarose, 0.5% and 2%, to encapsulate the enzymes as it has been previously shown that there is a significant difference in the pore sizes for these concentrations. Enzymes encapsulated in 2% gels were significantly more stable relative to enzymes encapsulated in 0.5% gels and solution phase enzymes under denaturing environments, indicating that increased protein confinement in higher percentage agarose gels led to greater enhancements in stability. Our data agrees well with previous investigations of the effects of molecular confinement on protein structure and function. Furthermore, as far as we know, this is the first report demonstrating clear dependence of function and stability of encapsulated proteins on the degree of confinement. We believe that this ability to control protein function by simply changing the hydrogel concentration will significantly impact a variety of industrial and biotechnological applications.

## Materials and Methods

### Materials

The enzymes, horseradish peroxidase (HRP), β-galactosidase (β-gal), and lysozyme were obtained from Sigma Aldrich (St. Louis, MO) and used without further purification. The enzyme substrates 2,2′-azinobis(3-ethylbenzthiazoline-6-sulphonate) (ABTS), hydrogen peroxide, and o-nitrophenyl β-D-galactoside (ONPG) were also purchased from Sigma Aldrich. Materials for preparation of the gels, agarose (molecular biology grade) was obtained from Bio-Rad Laboratories (Hercules, CA), alginic acid (sodium salt, low viscosity), and calcium chloride were purchased from Sigma Aldrich. Tris-HCl buffer was obtained from Life Technologies (Carlsbad, CA). 8-anilino-1-naphthalenesulfonic acid (ANS), guanidine hydrochloride (GdnHCl), and all other chemicals were purchased from Sigma Aldrich and used as received.

### Enzyme Encapsulation in Agarose Gels

The enzymes were encapsulated in agarose using standard entrapment techniques [16], [21], [22]. Briefly, solutions of agarose and enzyme in Tris-HCl buffer (100 mM, pH = 8.0) were combined at 45°C and the temperature was lowered to room temperature for solidification of agarose to encapsulate the enzyme. Stock agarose solutions were prepared by heating agarose/Tris-HCl buffer mixture at 90°C for 30 minutes. The agarose stock solutions were then cooled down to and maintained at 45°C. Freshly prepared enzyme stock solutions in Tris-HCl buffer were mixed with the agarose solutions at 45°C, aliquots of the agarose-enzyme solution were then immediately added to individual wells of a 96-well plate and then cooled to room temperature. Final agarose gel concentrations were either 2% or 0.5% w/v. Agarose-alginate hybrid gel-encapsulated enzyme formulations were prepared by first combining appropriate volumes of agarose, alginate, and enzyme stock solutions at 45°C. The mixture was then cooled to room temperature to solidify agarose, followed by the addition of 200 µL of 100 mM pH 8.0 Tris-HCl buffer containing 100 mM CaCl_2_ to crosslink the alginate. Final alginate concentrations in the hybrid gels were either 0.5% or 0.25% w/v.

### Kinetic Measurement of Enzyme Activity

The initial reaction rates of solution phase and gel-encapsulated HRP was determined using the ABTS assay; HRP mediates the oxidation of ABTS in the presence of H_2_O_2_ to form a soluble end product that can be monitored spectrophotometrically at 405 nm. HRP concentration for the initial rate measurements was 90 nM, and the ABTS and H_2_O_2_ concentrations were 100 µM and 40 µM, respectively. In order to estimate the Michaelis-Menten kinetic constants K_M_ and V_max_, the concentration of ABTS was varied between 0–300 µM, while keeping the concentrations of HRP and H_2_O_2_ constant, and the reaction rates were fitted to a non-linear regression model using GraphPad Prism. β-gal activities were measured by monitoring the hydrolysis of ONPG to o-nitrophenol at 420 nm; β-gal concentration was 10 nM and the ONPG concentration was 80 µM. The spectrophotometric measurements were made using a Tecan Infinite 200 PRO spectrophotometer (Durham, NC). Initial rates of ABTS oxidation and ONPG hydrolysis by agarose gels containing no enzyme were measured as controls. To determine enzyme activity under denaturing environments, solution phase and gel-encapsulated HRP formulations were equilibrated in the denaturant conditions for 5 minutes, followed by measurement of initial reaction rates. To check if HRP nonspecifically adsorbs to the agarose gels, 0.5% and 2% agarose gels were exposed to 90 nM HRP for 30 min followed by washing in Tris-HCl buffer for 10 min to remove any unbound or loosely bound enzyme. The activity of the HRP-exposed agarose gels were then measured and compared to agarose gels similarly exposed to Tris-HCl buffer containing no enzyme and agarose gels containing HRP.

### Determination of Enzyme Structure

Structural stability of the solution phase and gel-encapsulated enzymes was assessed using the ANS fluorescence assay. The fluorescence measurements were also carried out using the Tecan Infinite 200 PRO spectrophotometer (Durham, NC). The ANS fluorescence emission spectra of the enzyme formulations in standard Tris-HCl buffer conditions and denaturing conditions were collected after excitation at 360 nm. The final protein concentration was 40 µg/mL for all the measurements (HRP = 0.9 µM, β-gal = 0.09 µM, Lysozyme = 2.8 µM), and the ANS concentration was 13.5 µM. The solution phase and gel-encapsulated enzyme formulations were equilibrated in either Tris-HCl buffer or denaturant conditions for 5 minutes prior to the fluorescence measurements. Fluorescence spectra of the agarose or the agarose-alginate hybrid gels containing no enzyme were recorded similarly and subtracted from the spectra of enzyme. Furthermore, to check if the hydrogels interfered with the ANS measurements, fluorescence spectra were also measured for hydrogels containing the unfolded enzyme-ANS complex (prepared by exposing the unfolded protein to ANS in solution phase prior to gelation). The spectra for the unfolded enzyme-ANS complex in 0.5% and 2% agarose were statistically similar to that in Tris-HCl buffer, indicating no/minimal interference from agarose.

## Results

### Kinetic Parameters of HRP Encapsulated in Agarose Hydrogels

In order to study the role of degree of enzyme confinement on the kinetic parameters, we used agarose to encapsulate the model enzyme horseradish peroxidase (HRP) that catalyzes the oxidation of the substrate 2,2′-azinobis(3-ethylbenzthiazoline-6-sulphonate) (ABTS) in the presence of H_2_O_2_. Since significant differences in the average pore radii have been previously reported for 0.5% and 2% agarose gels (ca. 300 nm for 0.5% and ca. 75 nm for 2% agarose) [19], [20], we used these concentrations to generate agarose gel-encapsulated HRP formulations –0.5% AG-HRP and 2% AG-HRP. The initial rate V_0_ of agarose-encapsulated HRP relative to solution phase HRP was ca. 72% for 0.5% AG-HRP and ca. 58% for 2% AG-HRP, showing the clear dependence of enzymatic activity on the agarose concentration. Similar effects of agarose encapsulation on initial reaction rates were also observed for the unrelated enzyme β-galactosidase (β-gal); the initial rates of β-gal encapsulated in 0.5% and 2% agarose relative to solution β-gal were ca. 65% and ca. 54%, respectively. Lower values of initial rates for enzymes in agarose can be attributed to reduced rates of protein and substrate diffusion as described using kinetic models of heterogeneous catalysis. To better understand the role of encapsulation on enzyme kinetics, we performed experiments to calculate the apparent values of K_M_ and V_max_ for HRP encapsulated in agarose. Table 1 shows the values obtained for these parameters for HRP encapsulated in 0.5% and 2% agarose, as well as for solution phase HRP. We observed a slight decrease in the values of V_max_ and a significant increase in the K_M_ values for HRP encapsulated in agarose compared to those for solution phase HRP – especially for 2% AG-HRP. Therefore, these results suggest that the observed decrease in initial rates for agarose-encapsulated HRP can be attributed to diffusion-controlled kinetics of the encapsulated enzyme.

**Table 1:** Kinetic constants of ABTS oxidation by solution phase and agarose encapsulated HRP in buffer.

Condition	V_max_ (µM s^−1^)	K_M_ (µM)
Solution phase	10.3±0.3	4.7±0.2
0.5% agarose	8.6±0.7	6.4±0.4
2% agarose	7.2±0.6	9.1±0.6

The data are the averages of three replicates and the error represents the standard deviation.

### Effect of Agarose Gel Concentration on the Functional Stability of HRP

**Figure 1:**
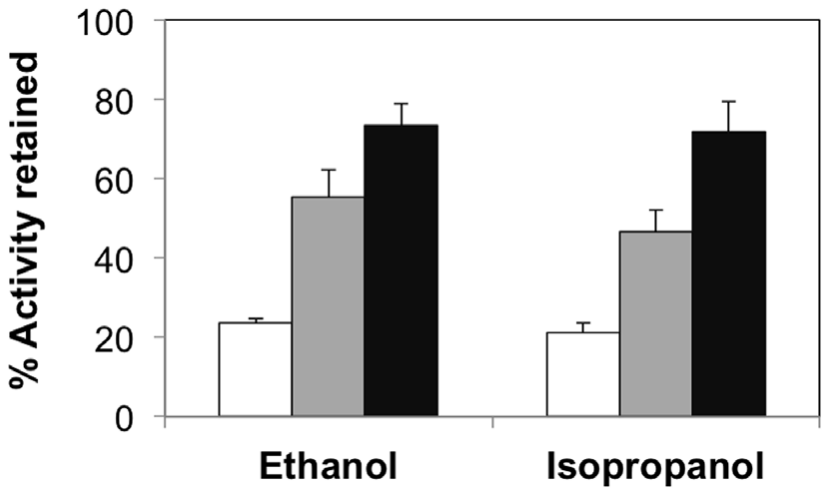
Functional stability of HRP encapsulated in agarose gels. Percent activity retained for solution phase HRP (white bars) and HRP encapsulated in 0.5% agarose (gray bars) and 2% agarose (black bars) in the presence of 35% ethanol and isopropanol. The relative activities were calculated by normalizing the activity in the presence of denaturants to the activity in buffer. Error bars indicate the standard deviation of triplicate measurements.

Having demonstrated the facile encapsulation of HRP in agarose and the effects of agarose concentration on enzyme kinetics under standard buffer conditions, we proceeded to study the influence of agarose concentration on the stability of the encapsulated enzymes under denaturing environments. For this, solution phase and encapsulated HRP were exposed to the denaturant – ethanol. Initially, we simply compared the initial rates for solution phase HRP and AG-HRP in standard buffer and buffer containing 35% ethanol. These studies revealed that while solution phase HRP only retained a small fraction of its initial intrinsic activity when exposed to ethanol (ca. 24%), HRP encapsulated in 0.5% (ca. 55%) and 2% agarose (ca. 73%) retained a higher percentage of their intrinsic activities (Figure 1). Similar enhancements in stability for agarose-encapsulated enzymes were also observed for β-gal formulations exposed to 35% ethanol (Figure S1) and HRP formulations exposed to 35% isopropanol (Figure 1). In addition, these results also indicate the role that degree of confinement has on the functional stability of HRP; specifically, HRP encapsulated in 2% agarose (highly confined) is significantly more tolerant to the denaturants compared to HRP encapsulated in 0.5% agarose (less confined). We performed supplementary experiments to confirm that molecular confinement, and not nonspecific protein adsorption to agarose, was responsible for the observed enhancements in enzyme stability in agarose. Specifically, we compared the activities of agarose films exposed to either buffer alone or buffer containing HRP. HRP-exposed agarose films showed negligible enzymatic activities, thereby suggesting that HRP does not nonspecifically adsorb to agarose; hence, nonspecific adsorption can be excluded as the underlying reason for the observed stabilization effects.

**Figure 2:**
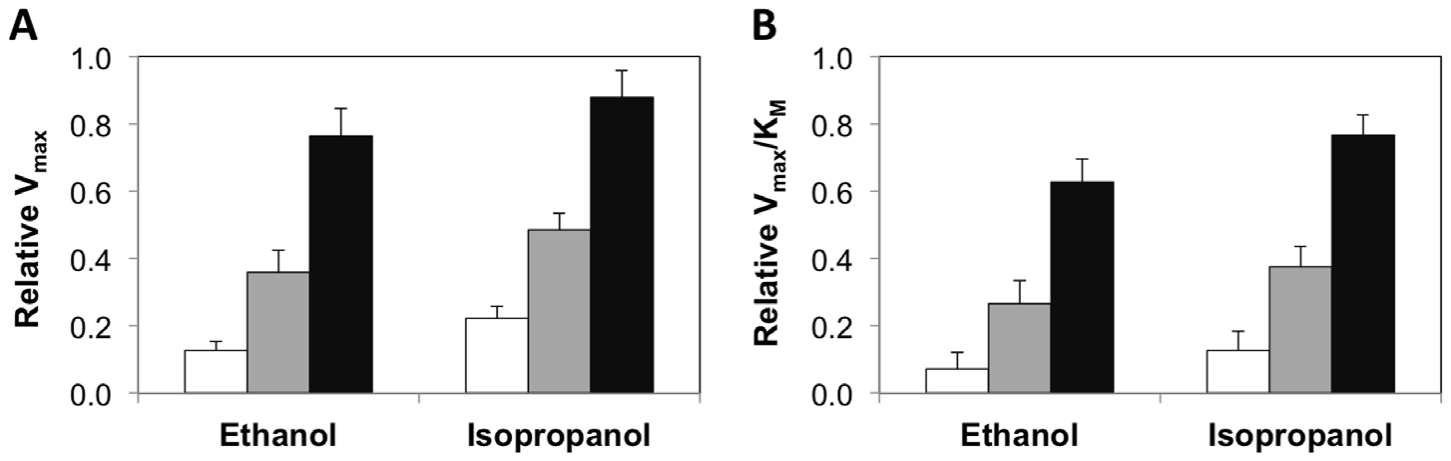
Kinetic constants of HRP catalysis in presence of denaturants. Relative kinetic constants of ABTS oxidation by solution phase HRP (white bars) and HRP encapsulated in 0.5% agarose (gray bars) and 2% agarose (black bars) in the presence of 35% ethanol and isopropanol – (a) V_max_ and (b) V_max_/K_M_. The relative kinetic constants were calculated by normalizing the kinetic parameters of HRP in the presence of denaturants to those in buffer. Error bars indicate the standard deviation of triplicate measurements.

We also calculated the kinetic parameters V_max_ and K_M_ for the AG-HRP formulations and solution phase HRP in the presence of the denaturants and compared them to the kinetic parameters obtained in standard buffer (Table 2, Figure 2). In all cases, there was an increase in the K_M_ values and a decrease in the values of V_max_ in the presence of the denaturants, consistent with previous observations of the effects on denaturation on enzyme kinetic parameters [23]–[25]. However, the effects of the denaturants on V_max_ and K_M_ were less evident for 2% AG-HRP when compared to 0.5% AG-HRP and solution phase HRP, thus supporting our initial observations of enhanced functional stability of HRP encapsulated in 2% agarose under denaturing conditions.

**Table 2:** Kinetic constants of ABTS oxidation by solution phase and agarose encapsulated HRP in 35% ethanol.

Condition	V_max_ (µM s^−1^)	K_M_ (µM)
Solution phase	1. 3±0.3	8.1±0.4
0.5% agarose	3.1±0.4	8.7±0.6
2% agarose	5.5±0.4	11.1±1.2

The data are the averages of three replicates and the error represents the standard deviation.

### Structural Stability of Agarose Encapsulated HRP

**Figure 3:**
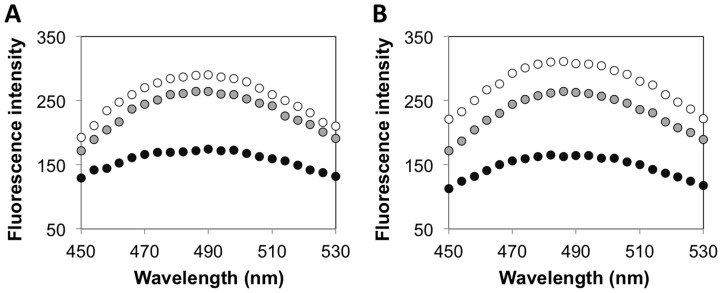
Structural stability of enzymes encapsulated in agarose gels. ANS fluorescence intensities (in arbitrary units) for solution phase enzyme (white) and enzyme encapsulated in 0.5% agarose (gray) and 2% agarose (black) exposed to 35% ethanol – (a) HRP and (b) β-gal. Fluorescence intensities for the agarose-protein formulations are reported after subtraction of the spectra of the corresponding agarose gels containing no enzyme.

To complement the kinetic assays, we conducted 8-anilino-1-naphthalenesulfonic acid or ANS-binding fluorescence assays to investigate the effect of denaturants on the structural stability of solution phase and encapsulated HRP. ANS is a fluorescent dye that has been used widely to probe conformational changes in proteins; binding of the ANS dye to the exposed hydrophobic regions of partially folded or fully unfolded proteins is accompanied by a marked increase in the fluorescence intensity and a blue shift of the peak maximum [26]–[29]. Consistent with previous studies using ANS fluorescence assays, when we exposed the various HRP formulations to the denaturants, we observed significant increase in the ANS signal, as well as a shift in the fluorescence emission peak to a broad maximum at 485 nm. Figure 3a shows the ANS fluorescence intensities of solution phase HRP and AG-HRP exposed to 35% ethanol. Significant increases in ANS fluorescence were observed for both solution phase HRP and 0.5% AG-HRP in 35% ethanol, whereas only a small increase was observed for 2% AG-HRP. We note that under the tested conditions, we did not observe an appreciable signal for the HRP formulations in standard buffer (data not shown). Supplementary experiments showed that the ANS signal for AG-HRP in ethanol disappeared when the concentration of ethanol is reduced to <5% (data not shown), indicating that the ethanol-induced denaturation of HRP encapsulated in agarose was reversible. The observed tolerance of 2% AG-HRP to ethanol was not limited to HRP, but was also observed for two other enzymes, β-gal and lysozyme (Figure 3b, Figure S2). We also conducted the ANS assays on HRP formulations in low pH buffers and buffers containing GdnHCl, and observed similar enhancements in structural stability for 2% AG-HRP, relative to native and 0.5% AG-HRP (Figure S3).

**Figure 4:**
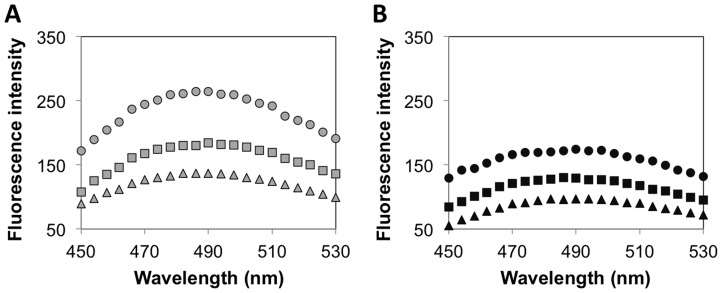
Structural stability of HRP encapsulated in agarose-alginate hybrid gels. ANS fluorescence intensities (in arbitrary units) for HRP encapsulated in agarose-alginate hybrid gels exposed to 35% ethanol – (a) 0.5% agarose (gray) and (b) 2% agarose (black) containing 0% (circles), 0.25% (squares), or 0.5% (triangles) alginate. Fluorescence intensities for the agarose-alginate-protein formulations are reported after subtraction of the spectra of the corresponding agarose-alginate gels containing no enzyme.

We performed additional experiments to confirm that the observed enhancements in the structural stability of 2% AG-enzyme formulations were indeed a result of increased protein confinement in 2% agarose relative to 0.5% agarose. For this, we exploited the ability of the naturally occurring polysaccharide alginate to form ionic crosslinked gels in the presence of divalent calcium ions. Changes in ANS fluorescence were measured for HRP encapsulated in agarose containing low and high amounts of alginate. If the increased protein confinement in 2% agarose gels contributed significantly to enhancements in stability, then HRP encapsulated in the hybrid gels should be more stable than HRP encapsulated in pure agarose gels. The specific test conditions were: HRP encapsulated in 0.5% and 2% agarose gels containing 0.25% or 0.5% alginate; 0.5% AG-HRP and 2% AG-HRP were used as controls. To prepare the agarose-alginate hybrid gel-encapsulated HRP formulations, agarose solutions containing HRP and alginate were allowed to gel first by lowering the temperature before the addition of calcium to achieve ionic crosslinking of alginate. As seen in Figure 4, the ANS signals for HRP encapsulated in agarose-alginate hybrid gels were lower than those of HRP encapsulated in pure agarose gels. Furthermore, the differences in the ANS signal of AG/Alg-HRP relative to AG-HRP were more pronounced for 0.5% agarose than observed for 2% agarose. We reason that the degree of confinement of the enzymes in 2% agarose was already high, and therefore the addition of alginate resulted in a smaller enhancement in protein stability relative to the enhancements in stability observed for 0.5% agarose-encapsulated enzymes.

## Discussion

Several experimental and theoretical reports support the prevailing concept that both confinement and crowding can potentially impact protein conformation. A variety of methodologies probing protein conformation have been employed to obtain mechanistic insights into the effects of different confining geometries, as well as the result of addition of inert macromolecules to simple dilute buffer solutions. These studies are significant since many protein functions, including enzymatic activity and signaling can be directly linked to protein conformation. It is also well appreciated that the conformational changes in a protein can contribute to its aggregation behavior [30]–[32]. Recent studies, albeit fewer, have also explicitly attempted to address the roles of crowding and confinement on protein stability under denaturing environments; these studies have revealed the ability of both confinement through encapsulation and crowding using inert macromolecules to enhance protein conformational and functional stability [7], [9], [14], [15], [18].

In our current study, we have investigated the effects of molecular confinement on functional and structural stability of proteins by encapsulating the model enzyme HRP in agarose gels. Since the porosity of agarose gels and therefore, the degree of protein confinement in agarose gels is directly related to the concentration of agarose, this system represents a simple model to study the effects of confinement on protein structure and function. Our data revealed a strong dependence of protein stability on the degree of confinement; HRP encapsulated in 2% agarose retained a greater percentage of enzyme activity and structure, relative to HRP encapsulated in 0.5% agarose and solution phase HRP, when exposed to the denaturants used in the study, ethanol and isopropanol. We hypothesize that the observed enhancements in stability are due to the lower porosity of 2% agarose gels relative to 0.5% agarose, which leads to a higher degree of protein confinement for HRP encapsulated in 2% agarose. As a test to the hypothesis, HRP was encapsulated in a hybrid agarose-alginate gel system, and we observed that an additional species, which would likely contribute to the protein confinement, could further enhance the structural stability of HRP. These results support the hypothesis that the increase in observed enhancements of protein stability for HRP in 2% agarose is due to an increase in the degree of confinement in higher percentages of agarose.

Data presented in this paper have two main outcomes. First, from a fundamental standpoint, the data provides a mechanistic insight into protein behavior in highly confined environments similar to those experienced by proteins inside living cells. The current work will guide our future experiments on the use of the agarose gel-based platforms to explore the effects on molecular confinement on the kinetics and extent of protein self-association and aggregation. This is of particular interest as current studies exploring protein association and aggregation under *in vivo*-like conditions have focused on the influence of macromolecular crowding on the underlying pathways and kinetics [4], [5], [33]. The effects of molecular confinement on these protein fates have been relatively unexplored.

Second, this study also contributes to the development of applications that use encapsulated proteins; examples include drug delivery platforms, biosensors, and biofuel cells [34]–[36]. Encapsulated proteins could also potentially be used for the development of enzyme bioreactor-based medical devices to assist patients with kidney and/or liver disease [35], [37]. These applications rely on the long-term operational stability of the encapsulated proteins, and therefore enhanced stability of proteins encapsulated in agarose as demonstrated in this paper is of direct relevance to the applicability of proteins in the biotechnology and biomedical industries.

In conclusion, our results presented here demonstrate the role of encapsulation in agarose gels on protein function, structure, and stability. Analysis of the kinetic constants under standard buffer conditions suggested that encapsulation of HRP in agarose was facile and did not lead to significant protein denaturation, and the enzyme kinetics for agarose-encapsulated HRP was diffusionally limited. When exposed to denaturing environments, agarose-encapsulated HRP retained a higher percentage of its structure and function relative to the solution phase enzyme. Furthermore, HRP encapsulated in 2% agarose (highly confined) is significantly more resistant to unfolding compared to HRP encapsulated in 0.5% agarose (less confined). Finally, similar trends were also observed for two other structurally and functionally different enzymes indicating the generality of the observed phenomenon. The observed enhancements in stability of enzymes encapsulated in agarose will be potentially useful in applications ranging from sensing and diagnostics to drug delivery and medical devices.

## Supporting Information

Figure S1 **Functional stability of β-gal encapsulated in agarose gels.** Percent activity retained for solution phase β-gal (white bars) and β-gal encapsulated in 0.5% agarose (gray bars) and 2% agarose (black bars) in the presence of 35% ethanol. The relative activities were calculated by normalizing the activity in the presence of 35% ethanol to the activity in buffer. Error bars indicate the standard deviation of triplicate measurements.
(TIFF)

Figure S2 **Structural stability of lysozyme encapsulated in agarose gels.** ANS fluorescence intensities (in arbitrary units) for solution phase lysozyme (white) and lysozyme encapsulated in 0.5% agarose (gray) and 2% agarose (black) exposed to 35% ethanol. Fluorescence intensities for the agarose-lysozyme formulations are reported after subtraction of the spectra of the corresponding agarose gels containing no enzyme.
(TIFF)

Figure S3 **Structural stability of HRP encapsulated in agarose gels.** ANS fluorescence intensities (in arbitrary units) for solution phase enzyme (white) and enzyme encapsulated in 0.5% agarose (gray) and 2% agarose (black) exposed to (a) low pH (pH = 2.8) and (b) 4.7 M GdnHCl. Fluorescence intensities for the agarose-HRP formulations are reported after subtraction of the spectra of the corresponding agarose gels containing no enzyme.
(TIFF)
