# Supplementary figures and images for: Function, Structure, and Stability of Enzymes Confined in Agarose Gels
Source: PLoS One. 2014 Jan 21;9(1):e86785. doi: 10.1371/journal.pone.0086785 (PMC3897775; doi:10.1371/journal.pone.0086785)

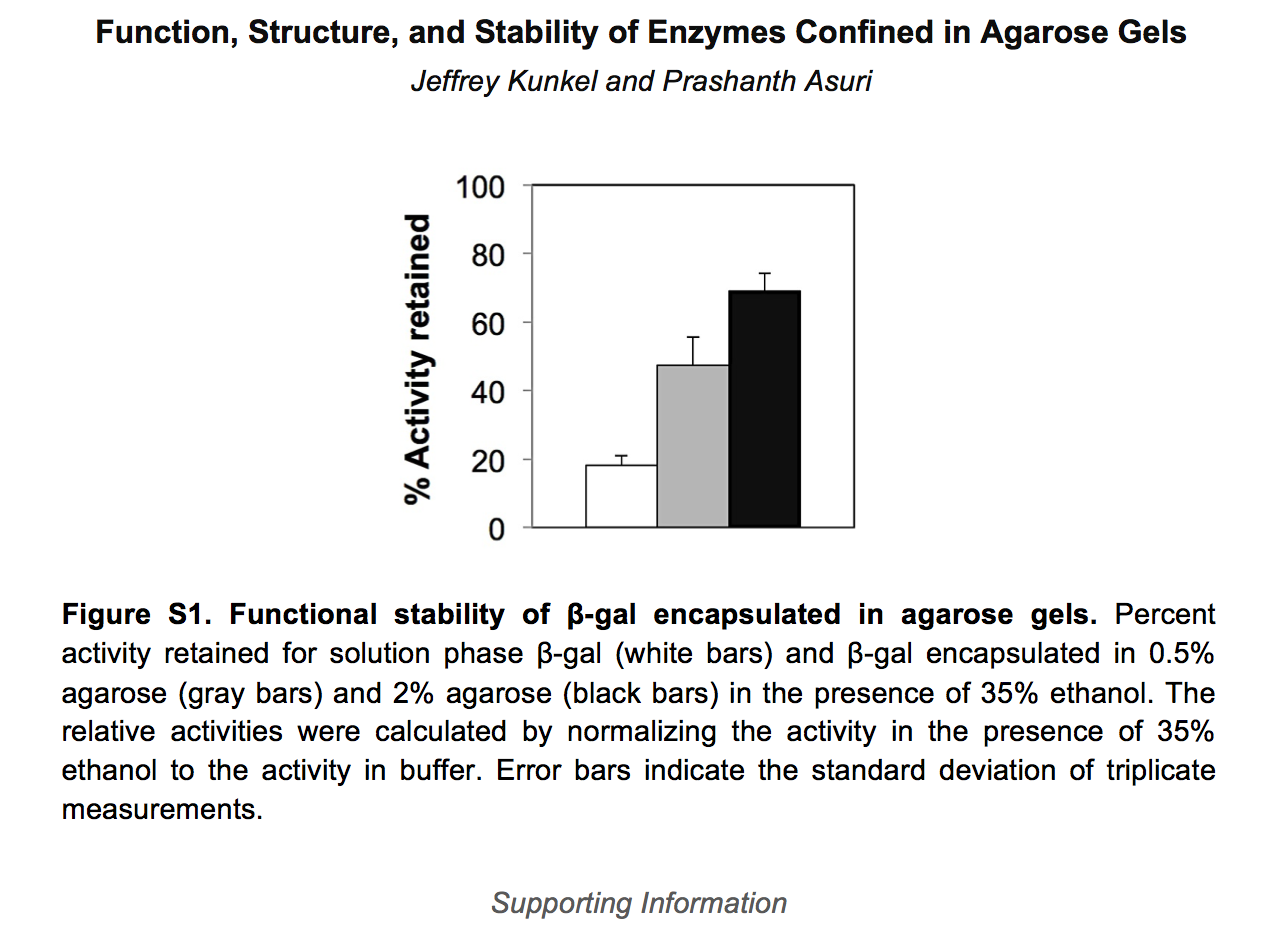

Supplement: Figure S1 — Functional stability of β-gal encapsulated in agarose gels. Percent activity retained for solution phase β-gal (white bars) and β-gal encapsulated in 0.5% agarose (gray bars) and 2% agarose (black bars) in the presence of 35% ethanol. The relative activities were calculated by normalizing the activity in the presence of 35% ethanol to the activity in buffer. Error bars indicate the standard deviation of triplicate measurements. (TIFF) [file pone.0086785.s001.tiff]

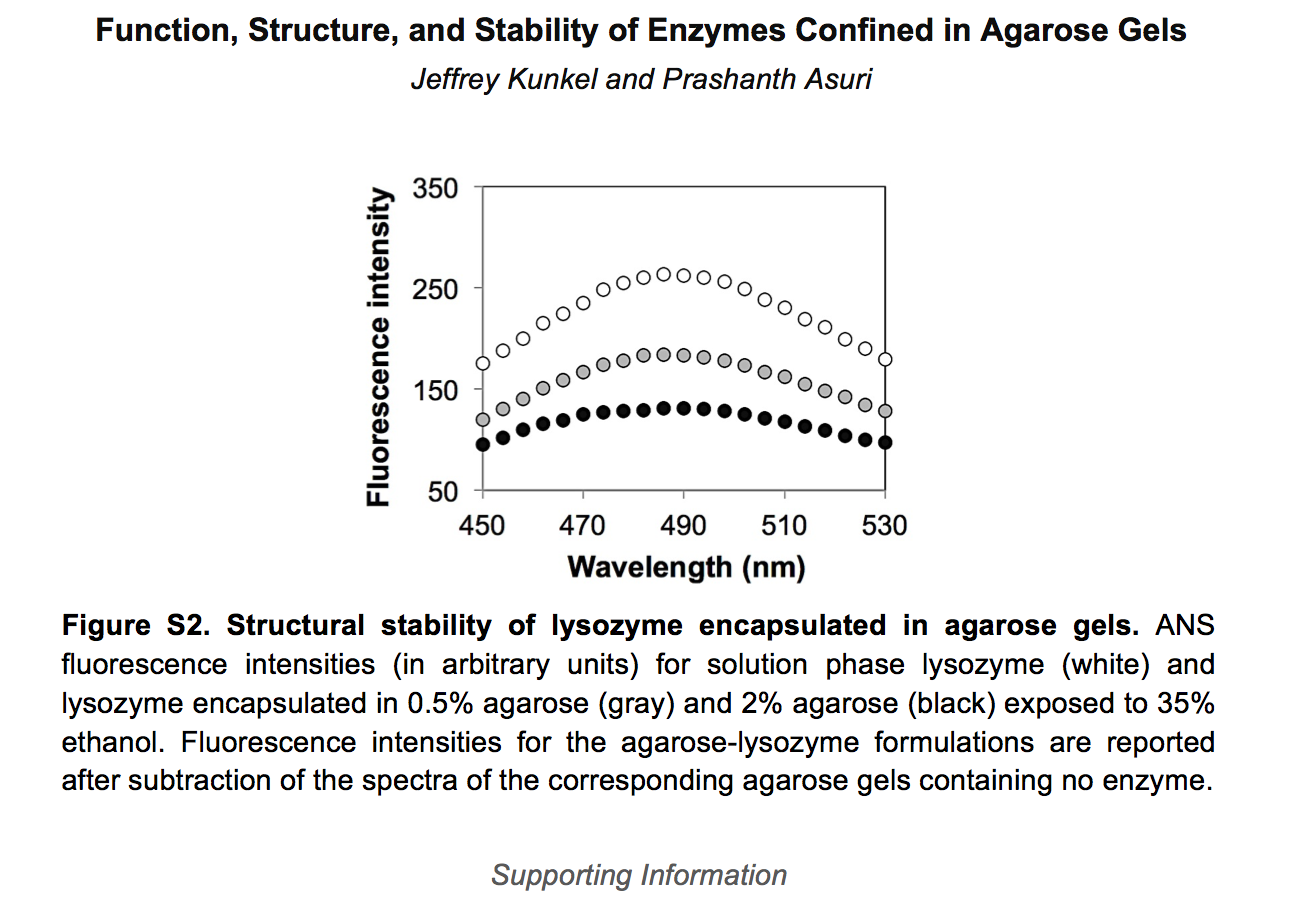

Supplement: Figure S2 — Structural stability of lysozyme encapsulated in agarose gels. ANS fluorescence intensities (in arbitrary units) for solution phase lysozyme (white) and lysozyme encapsulated in 0.5% agarose (gray) and 2% agarose (black) exposed to 35% ethanol. Fluorescence intensities for the agarose-lysozyme formulations are reported after subtraction of the spectra of the corresponding agarose gels containing no enzyme. (TIFF) [file pone.0086785.s002.tiff]

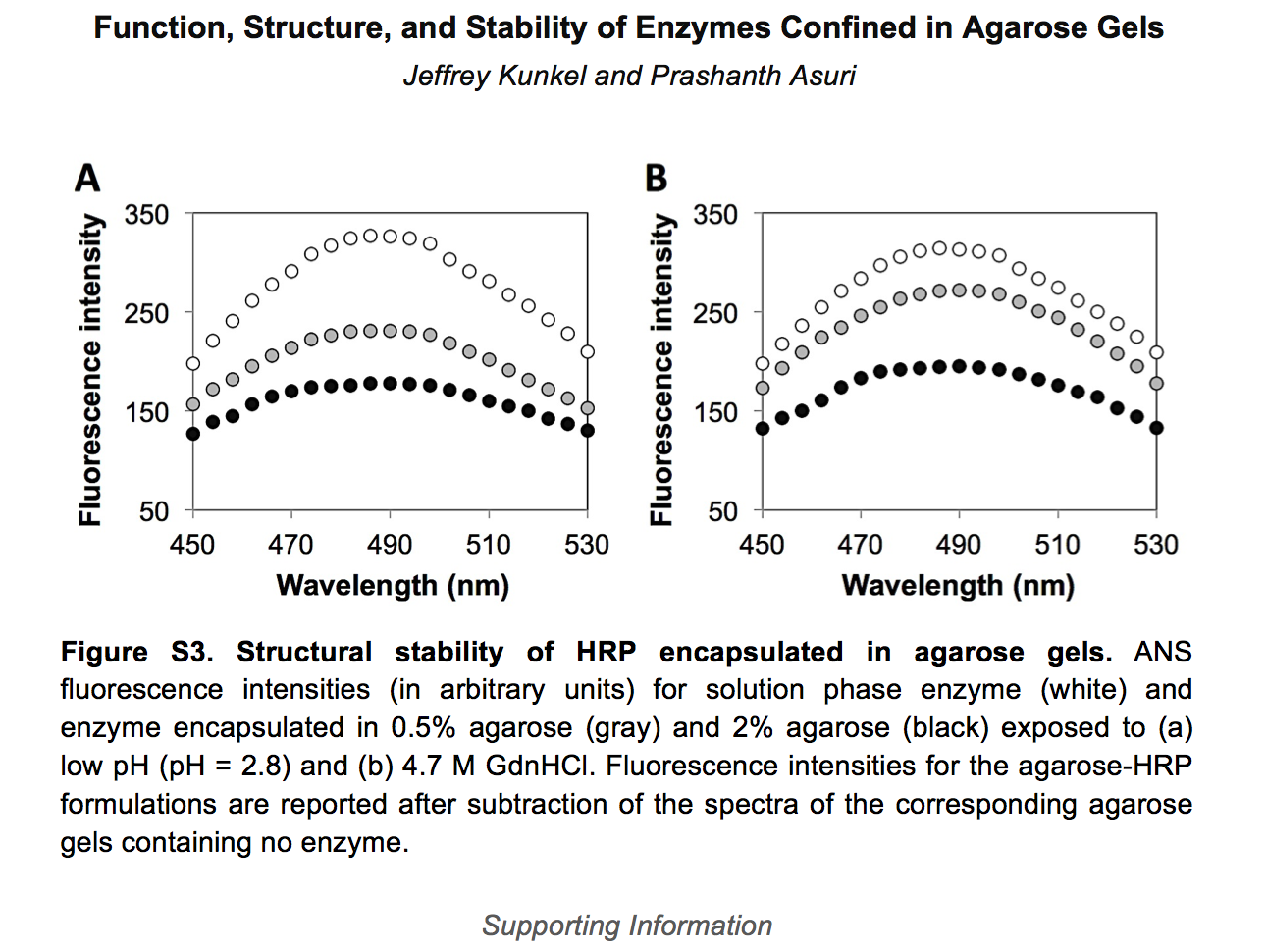

Supplement: Figure S3 — Structural stability of HRP encapsulated in agarose gels. ANS fluorescence intensities (in arbitrary units) for solution phase enzyme (white) and enzyme encapsulated in 0.5% agarose (gray) and 2% agarose (black) exposed to (a) low pH (pH = 2.8) and (b) 4.7 M GdnHCl. Fluorescence intensities for the agarose-HRP formulations are reported after subtraction of the spectra of the corresponding agarose gels containing no enzyme. (TIFF) [file pone.0086785.s003.tiff]
